# Glucose and glycogen affects Ca^2+^ transient during fatigue to a greater extent in the least than in the most fatigue resistant mouse FDB fibers

**DOI:** 10.14814/phy2.70065

**Published:** 2024-10-16

**Authors:** Erik Hesse, Tarek Ammar, Jean‐Marc Renaud

**Affiliations:** ^1^ Department of Cellular and Molecular Medicine University of Ottawa Ottawa Ontario Canada

**Keywords:** fatigue, FDB, tetanic intracellular calcium, unstimulated intracellular calcium

## Abstract

The overall objective was to determine how no extracellular glucose and/or low glycogen content affect fatigue kinetics in mouse flexor digitorum brevis (FDB) single muscle fibers. High glycogen content (Hi GLY), near normal in situ level, was obtained by incubating fibers in culture medium containing glucose and insulin while low glycogen content (Lo GLY), at about 19% of normal in situ level, was achieved by incubating fibers without glucose. Neither Lo GLY nor the absence of extracellular glucose (0GLU) affected tetanic [Ca^2+^]_i_ prior to fatigue. The number of contracting unfatigued fibers versus stimulus strength relationship of Lo GLY‐0GLU fibers was shifted to higher voltages compared to Hi GLY fibers exposed to 5.5 mM glucose (5GLU). The relationship for Lo GLY‐0GLU fibers was shifted back toward that of Hi GLY‐5GLU fibers when glucose was reintroduced, whereas the removal of glucose from Hi GLY‐5GLU fibers had no effect. Fatigue was elicited with one 200 ms long tetanic contraction every s for 3 min. Both Lo GLY and 0GLU increased the rate at which intracellular tetanic concentration ([Ca^2+^]_i_) declined and unstimulated [Ca^2+^]_i_ increased during fatigue in the order of the least fatigue resistant > mid fatigue resistant > the most fatigue resistant fibers.

## 1 INTRODUCTION

Muscle fatigue is defined as a decreased capacity of skeletal muscle to generate force or do work when repetitively stimulated and is considered a protective mechanism to preserve cellular integrity by preventing damaging ATP depletion and/or large increases in [Ca^2+^]_i_ (McKenna et al., 2008; Renaud et al., 2023). A major cause for the decrease in force/work involves a decrease in the amount of Ca^2+^ released by the sarcoplasmic reticulum resulting in submaximal [Ca^2+^]_i_ and incomplete activation of the contractile components (Chin & Allen, 1997). The mechanism for the decreased Ca^2+^ release is not fully understood but in part involves a decrease in membrane excitability as recently reviewed (Renaud et al., 2023). Several membrane components that control membrane excitability during contraction and fatigue, that is, Na^+^ K^+^ ATPase pump (NKA), ATP‐sensitive K^+^ channels (K_ATP_ channel) and Ca^2+^ ATPase pump (SERCA), depend on glycolytic ATP generation (Dhar‐Chowdhury et al., 2005, 2007; Okamoto et al., 2001; Weiss & Lamp, 1989; Xu et al., 1995). As a consequence of this ATP dependence, one expects that glycogen and extracellular glucose availability affects fatigue kinetics.

A positive relationship exists between initial glycogen content and work time in toad and human; that is, the higher the initial glycogen content the longer the time a work load is maintained (Bergström et al., 1967; Kabbara et al., 2000). Human became unable to exercise when glycogen content reached extremely low values; that is, 15–30 μM/g dry weight (Hermansen et al., 1967). Ca^2+^ release capacity of mechanically skinned fiber in response to transverse tubule depolarization or action potential is reduced as the initial glycogen content is depleted (Stephenson et al., 1999; Watanabe & Wada, 2019). Depleting glycogen stores with one fatigue bout followed by a recovery in the absence of extracellular glucose to prevent glycogen replenishment results in faster rate of tetanic [Ca^2+^]_i_ and force decrease during a second fatigue bout compared when compared to muscles that had been allowed to recover their glycogen store (Chin & Allen, 1997; Helander et al., 2002).

In rat and human, glucose ingestion or infusion prolonged exercise time (Coggan & Coyle, 1987; Coyle et al., 1986; Slentz et al., 1990). Glucose infusion reduced the extent of the force decrease during fatigue in rat plantaris and soleus muscles in situ (Karelis et al., 2002; Marcil et al., 2005). Although in the later two studies insulin level was high, the glucose effect was not observed under a hyperinsulinemic‐euglycemic condition (Karelis et al., 2003). The decrease in tetanic force of rat soleus during a 120 s long tetanic (60 Hz, 30°C) was significantly faster and greater in the presence of 2‐deoxyglucose, to inhibit glycolysis (Murphy & Clausen, 2007).

In some of the above studies, glycogen content was modified using different diets, raising the possibility that some of the muscle performance was influenced by hormonal changes prior to exercise. In other studies, glycogen was depleted by a series of contraction bouts in the absence of glucose, a protocol that fails to differentiate between the effects of low glycogen content and no extracellular glucose. Finally, when glycogen content was depleted by one fatigue bout followed by recovery in the absence of glucose; the second fatigue bout was triggered even though tetanic [Ca^2+^]_i_ and force had not fully recovered; that is, muscles were still in a fatigue state. Finally, some studies were carried out with different muscles with different fiber type compositions. However, it remains unknown how various fiber types are affected by glycogen content and extracellular glucose.

The overall objective was to determine how low glycogen content and/or no extracellular glucose affect fatigue kinetics in mouse FDB single muscle fibers. At 37°C, three major fatigue kinetics are observed from single FDB fibers: (i) fatigue resistant fibers with the slowest decrease in tetanic [Ca^2+^]_i_, which remains above pre‐fatigue level at the end of a 3 min fatigue bout; (ii) fatigable fibers with the greatest and fastest decrease in tetanic [Ca^2+^]_i_; (iii) mid‐fatigue resistant fibers with intermediate decreases in tetanic [Ca^2+^]_i_ (Selvin & Renaud, 2015). So, for this study, FDB single fibers were separated by trituration after a collagenase treatment to test several fibers per experiment allowing us to have sufficient number of fibers for each experimental condition and fiber type. Force could not be measured from these fibers. However, it is well established that a major cause for the force decrease during fatigue is less Ca^2+^ release resulting in submaximal activation of contractile components (Chin & Allen, 1997); that is, in this study fatigue kinetics were followed from changes in tetanic [Ca^2+^]_i_. High and low glycogen contents were achieved by incubating FDB single fibers in culture media with and without glucose, respectively. We hypothesize that both low glycogen content and no extracellular glucose accelerate the decrease in tetanic [Ca^2+^]_i_ during fatigue in the order of the least fatigue resistant > mid fatigue resistant > the most fatigue resistant fibers.

## 2 METHODS

### 2.1 Animals, muscle bundles and single fiber preparation

Experiments were carried out using FDB muscle bundles and single fibers from flexor digitorum brevis (FDB) muscles from CD‐1 mice (Charles River, Canada). Mice were 2–4 months in age and weighed 20–30 g. Mice were fed ad libitum with Teklad global 18% protein rodent diet (Cat. #T.2018.15, Envigo Rms, Llc, Canada) and housed according to the guidelines of the Canadian Council for Animal Care (CCAC). The Animal Care Committee of the University of Ottawa approved all experimental procedures used in this study. Mice were anesthetized with a single intraperitoneal injection of 2.2 mg ketamine (Cat. #7087, CDMV, Canada), 0.44 mg xylazine and 0.22 mg acepromazine (Cat. #118550, CDMV, Canada) per 10 g of body mass (Cat. #1047, CDMV, Canada). Mice were then sacrificed by cervical dislocation.

### 2.2 FDB single muscle fibers

Single fibers were isolated from FDB muscles as previously described (Selvin et al., 2015). Briefly, FDB muscles were incubated 3 h at 37°C in minimum essential medium (MEM) with Earle's salt and L‐glutamine (Cat. # 11095080, Gibco, Canada) supplemented with Gibco heat inactivated 10% (v/v) foetal bovine serum (FBS, Cat. # A5209401, ThermoFisher, Canada,), 0.2% (w/v) collagenase type I (Cat. # LS004197, Worthington, USA), 100 units/mL of penicillin and 100 μg/mL of streptomycin (Cat. # 15140122, ThermoFisher, Canada). Fibers were separated by gentle trituration in 3 mL collagenase‐free MEM. One hundred μL of concentrated fiber‐containing medium was placed on a Matrigel (Cat. # 47743–718, VWR, Canada) pre‐coated 12 mm diameter coverslip (Cat. # 89015–725, VWR, Canada).

#### 2.2.1 Glycogen lowering protocol

Single fibers were divided into three groups. One group was incubated in MEM culture medium supplemented with 10% FBS and a second group with the addition of 300 μU/mL insulin (Cat. #NC1674236, Sigma, Canada) to allow for the highest possible glycogen content, here defined as the Hi GLY condition. The third group of fibers were incubated in zero glucose Dubelco's modified Eagle medium (DMEM, Cat. # 11966025, ThermoFisher, Canada) supplemented with 300 μU/mL insulin to allow for glycogen depletion, here defined as the Lo GLY condition. Incubations were all at 37°C for a minimum of 1 h and maximum of 3 h before fibers were used for contractile threshold and [Ca^2+^]_i_ measurements. Measurements were carried out the day fibers had been prepared because stimulation threshold and number of contracting fibers remained constant for at least 3 h after being transferred to the experimental chamber, whereas an overnight incubation results in fibers with greater stimulation threshold and in many cases becoming unexcitable (Selvin et al., 2015).

#### 2.2.2 Physiological solutions and experimental temperature

Coverslips containing single FDB fibers were mounted into a 370 μL chamber (model RC‐25, Warner Instruments, USA). Fibers were continuously perfused in physiological solution with a flow rate of 5 mL/min; the physiological solution containing (mM): 118.5 NaCl, 4.7 KCl, 2.4 CaCl_2_, 3.1 MgCl_2_, 25 NaHCO_3_, 2 NaH_2_PO_4_, with or without 5.5 D‐glucose and 0.2% FBS (v/v). The FBS was added to prevent (i) complete loss of contractility and (ii) to lower stimulation threshold over time especially when fibers are stressed with a fatigue bout (Selvin et al., 2015). Solutions were continuously bubbled with 95% O_2_–5% CO_2_ and had a pH of 7.4. The experimental temperature was simultaneously controlled by heating the plate to which the chamber was mounted, and the physiological solution running through the chamber, using a dual channel heater controller (model TC‐344B, Warner Instruments, USA). The fiber temperature was about 22°C after positioning the RC‐25 chamber on the microscope. Temperature was increased to 37°C at a rate of 2°C every 100 s to prevent loss of fiber contractility (Selvin et al., 2015).

#### 2.2.3 Stimulation protocol

Tetanic contractions were elicited using field stimulation. Two platinum electrodes were positioned along each side of the RC25 chamber. The electrodes were connected to a Grass S88X stimulator and Grass stimulation isolation unit (Grass Technologies, West Warwich, RI, and USA). Tetanic stimulation consisted of 200 ms trains of 0.3 ms 10 V pulses at a frequency of 140 Hz. Prior to fatigue, tetanic contractions were elicited every 100 s and for fatigue every 1 s for 3 min.

#### 2.2.4 Contraction threshold measurements

Fibers were allowed an initial 30 min equilibrium after being transferred to the fiber chamber. Fibers were divided into four experimental conditions: (i) Hi GLY/5GLU were fibers incubated in glucose‐containing MEM culture medium and tested in 5.5 mM glucose containing physiological solution; (ii) Hi GLY/5 to 0GLU were fibers incubated in glucose‐containing MEM culture medium, equilibrated in glucose‐containing physiological solution but tested 10 min after removing extracellular glucose; (iii) Lo GLY/0GLU were fibers incubated in glucose‐free DMEM culture medium and tested in glucose‐free physiological solution; (iv) Hi GLY/0 to 5GLU were fibers incubated in glucose‐free DMEM culture medium, equilibrated in glucose‐free physiological solution, but tested 10 min after adding 5.5 mM glucose. Changes in contraction threshold were measured by increasing the stimulus strength from 3 to 15 V stepwise by 1 V. For each stimulus strength, we counted the number of fibers that shortened. V_50_, defined as the stimulus strength at which 50% of fibers contracted, was calculated using non‐linear regression analysis to a sigmoidal curve (SigmaPlot, USA, Version 13) as follows:

$$\#\text{Contracting Fibers}=\frac{{\mathrm{CT}}_{\max}}{1+e^{\left(\frac{-\left(V-V50\right)}{C}\right)}}$$

where, CT_max_: Maximum number of contracting fibers. *V*: Stimulation strength. *C*: Constant.

Fibers from five mice were used for each experimental condition. Regression analyses were carried for each mouse and experimental condition allowing the analysis to provide a value for each of CTmax, V_50_ and the constant “*C*.” Only the mean ± SD of V_50_ is provided in the Results section.

#### 2.2.5 [Ca^2+^]_i_ measurements

Fibers were loaded with Fura2 by incubating fibers 30 min at 37°C in culture medium containing 5 μM Molecular Probes Fura‐2 am (Cat. # F1201, ThermoFisher, Canada). Fura‐2 was alternatively excited at wavelengths of 340 and 380 nm, and light emission was measured at 505 nm. Alternating between the different excitation wavelengths and fluorescence measurement were carried out using the IonOptix dual fluorescent contractility device (USA) employing the following filters: 340 ± 12, 380 ± 6, and 505 ± 6 nm. Data acquisition was set at 200 Hz; the 505 nm light emitted in 2 ms during excitation at 340 nm was divided by the 505 nm light emitted in 2 ms during excitation at 380 nm. [Ca^2+^]i was then calculated from the ratio as previously described (Cifelli et al., 2007) using the following equation:

$$\left[{\mathrm{Ca}}^{2+}\right]i=\mathrm{Kd}\times \frac{\left(R-R_{\min}\right)}{\left(R_{\max}-R\right)}\times \beta$$

Kd is the dissociation constant of fura‐2 for Ca^2+^, being 224 nM at 37°C (Li et al., 2008), RMIN being 89 ± 0.7% of the ratio measured in resting fibers, RMAX being 126.1% ± 7.5% of the ratio during a tetanic contraction under control conditions, and *β* the fluorescence at 380 nm excitation of Ca^2+^‐free divided by Ca^2+^‐bound Fura‐2, being 3.17 ± 0.72. Measurements of RMIN were carried out by exposing fibers to a physiological solution containing no Ca^2+^, 1 mM EGTA, a Ca^2+^ buffer, and ionomycin (Cat. # I24222, Sigma, Canada), a Ca^2+^ ionophore, in order to reduce [Ca^2+^]i to the lowest possible level. RMAX was measured by exposing fibers to a solution containing 10 μM Ca^2+^ and ionomycin.

In this study, it is not possible to determine the fiber type at the time of [Ca^2+^]_i_ measurements, we made [Ca^2+^]_i_ measurements until there were enough fibers for each fiber type and physiological condition. As a consequence, the numbers of tested fibers differ between physiological conditions and the three groups of fatigue kinetic.

#### 2.2.6 Fatigue: Glycogen and glucose conditions

Fibers were divided into four experimental conditions. Hi GLY/5GLU condition for which fibers were incubated in glucose containing MEM culture medium to maintain a high glycogen content and tested in a physiological solution containing 5.5 mM glucose. Hi GLY/0GLU condition for which fibers with high glycogen content were initially superfused with glucose containing physiological solutions and with the removal of glucose 10 min prior to the fatigue bout. Lo GLY/5GLU condition for which fibers were incubated in zero glucose DMEM culture medium to lower glycogen content, initially superfused in zero glucose physiological solution and with the addition of 5.5 mM glucose 10 min prior to fatigue. Lo GLY/0GLU condition for which fibers with low glycogen content were tested with zero glucose physiological solution.

## 3 GLYCOGEN MEASUREMENTS

Glycogen content was measured in both muscle bundles and single fibers. Muscle bundles were frozen clamped using metal clamps pre‐cooled in liquid nitrogen immediately after dissection, prior to or after a fatigue bout. For single fibers, glycogen was determined after various times of incubation following trituration. One mL of fiber‐rich collagenase‐free suspension was centrifuged 10 s at 600*g*. Fibers from the pellet were washed in an ice‐cold glucose free physiological solution and centrifuged a 2nd time. The pellet was flash frozen directly in the centrifuge tube by pressing it into a metal casing pre‐cooled in liquid nitrogen with a metal rod pushed on the pellet‚ also pre‐cooled in liquid nitrogen. Muscle bundles and fibers were stored at −80°C until glycogen determination.

Muscle bundles and single fibers were freeze‐dried overnight using a lyophilizer (Freezemobile 6, Virtis, USA). Lyophilized fibers from bundles were separated from tendons where single fibers were separated from crystalized ions before being weighed on a 6 decimal analytical balance (Mettler Toledo, XS105, USA). Lyophilized muscle tissue was added to 66 μL of 1 N NaOH and incubated at 80°C for 15 min to degrade endogenous glucose. The NaOH solution was then diluted by adding 198 μL of double distilled H_2_O. Sixty‐six μL of the diluted solution was added to 132 μL of 0.15 M sodium acetate buffer. Glycogen was hydrolyzed to β‐D‐glucose by adding 6 μL of 260 U/mL amyloglucosidase (Cat. # 10115, Sigma, USA) and incubated at 30°C for 2 h. β‐D‐glucose was specifically oxidized to D‐glucono‐δ‐lactone by the addition of glucose oxidase (Cat. #700485, Cayman Chemical, USA) forming hydrogen peroxide in the process. Hydrogen peroxide, in the presence of horseradish peroxidase (Cat. #700485, Cayman Chemical, USA), reacts with 10‐acetyl‐3,7‐dihydroxyphenoxazine (ADHP, Cat. #10010469, Cayman Chemical, USA) in a 1:1 stoichiometry to generate the highly fluorescent product resorufin which was measured with a plate reader (Perkin Elmer, Model LS50B, USA) using an excitation wavelength of 535 nm and measuring fluorescence at 590 nm.

## 4 FDB BUNDLES: TETANIC FORCE MEASUREMENTS

FDB bundles, 1–2 mg, were prepared as previously described (Cifelli et al., 2007). Briefly, fibers controlling the 4th digit were excised by cutting along and very close to the lateral fascia separating the fibers of the 3rd and 4th digit, cutting away fibers of the 3rd digit from the facia. FDB bundles were transferred to a chamber made of Plexiglas measuring 10 mm long, 10 mm wide, and 5 mm deep. Solution entered the chamber through two Teflon pieces located just behind the bundles. The total flow of solution was 15 mL/min divided equally over the upper and lower surface of the bundle. One end of the bundle was attached to a force transducer (model 400A; Aurora Scientific Canada) and the other end to a fixed metal pin.

### 4.1 Physiological solutions, experimental temperature, stimulation, and fatigue protocol

FDB bundles were superfused with the same physiological solution used for single fibers, except that no FBS was added. Solutions were continuously bubbled with 95% O_2_–5% CO_2_ and had a pH of 7.4. Solution was heated to 37°C just before entering the muscle chamber. Tetanic contractions were elicited with field electrical stimulations, 200 ms trains of 0.3 ms, 10 V at a frequency of 200 Hz, applied across two transversely located platinum wires (4 mm apart) located on opposite sides of the bundle. Platinum electrodes were connected to a Grass S88X stimulator and a Grass stimulation isolation unit (Grass Technologies, West Warwich, RI, and USA). Prior to fatigue tetanic contractions were elicited every 100 s and for fatigue every 1 s for 3 min.

### 4.2 Tetanic force measurements

FDB bundle length was adjusted to give maximum tetanic force. Bundles were then allowed to equilibrate 30 min in either the absence or presence of 300 μU/mL insulin; for one group of bundles, glucose was removed 10 min prior to the fatigue bout. The force transducer was connected to a data acquisition system (KCP13104; Keithley), and data were recorded at 5 kHz. Two parameters were measured using a computer program to analyze the contraction waveforms. Tetanic force, defined as the force developed while bundles were electrically stimulated, was calculated as the difference between the maximum force during a contraction and the baseline force measured 5 ms before stimulation. Unstimulated force, defined as the force measured between contractions, was calculated as the difference between the baseline 100 ms prior to a contraction and the baseline after bundle length had been adjusted.

## 5 DATA AVAILABILITY AND STATISTICAL ANALYSIS

The data that support the findings of this study are available from the corresponding author upon reasonable request. Data are presented as mean ± standard deviation (SD). For FDB bundles, the number of samples represents the number of bundles tested. For single fibers, the number of samples represents the number of fibers tested/ the number of mice used. Significant differences between V_50_ values were tested using *t*‐test as there were only four means. Split plot ANOVA designs were used to determine significant differences for all experiments involving different times of data collections. Glycogen and glucose conditions were in the whole plot and the time condition in the split plot. ANOVA calculations were made using the Version 9.3 GLM (General Linear Model) procedures of the Statistical Analysis Software (SAS Institute Inc., Cary, NC USA). When a main effect or an interaction was significant, the least square difference (L.S.D.) was used to locate the significant differences (Steel & Torrie, 1980). The word “significant” refers only to a L.S.D. statistical difference for *p* < 0.05.

## 6 RESULTS

### 6.1 Single FDB fibers

#### 6.1.1 Glycogen content

**FIGURE 1:**
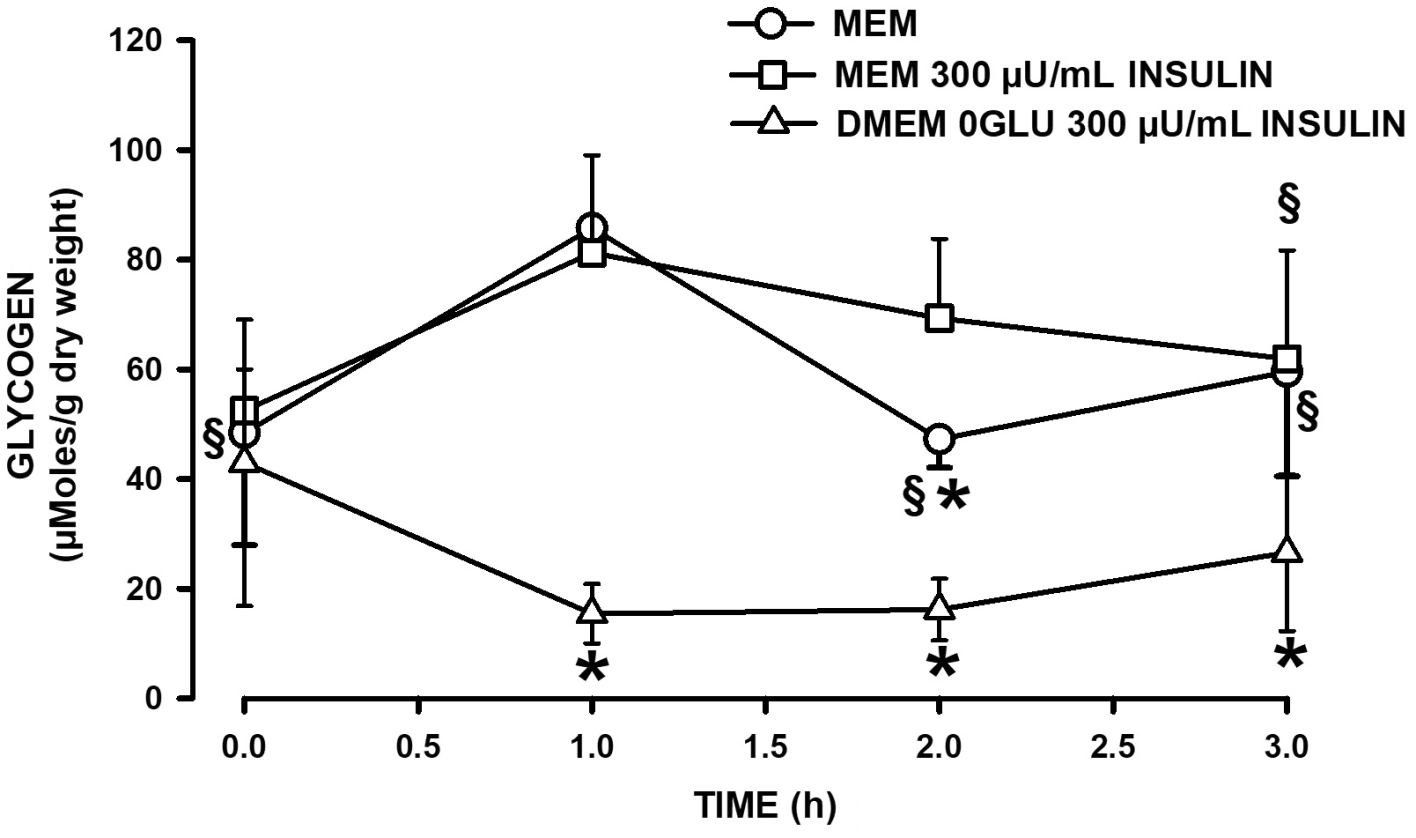
Insulin is necessary to maintain high glycogen content while a lack of extracellular glucose depleted glycogen reserves in single FDB fibers. Glycogen was measured in single FDB fibers at various times. FDB muscles were treated 3 h with collagenase in MEM supplemented with 10% FBS and after trituration FDB fibers were incubated at 37°C in MEM supplemented with 10% FBS in the absence (○, MEM), in the presence of 300 μU/mL insulin (󠇧□, MEM 300 μU/mL insulin) or in glucose‐free DMEM supplemented with 10% FBS and 300 μU/mL insulin (Δ, DMEM 0GLU 300 μU/mL insulin). Vertical bars represent the SD of the following mice number: 3 for MEM, 9 for MEM 300 μU/mL insulin and 7 for DMEM 0GLU 300 μU/mL insulin. ANOVA: GLY‐GLU condition × Time interaction *p* = 0.012. ^§^Mean glycogen content significantly different from that at 1 h.; *Mean glycogen content significantly different from that of MEM 300 μU/mL insulin;, L.S.D. *p* < 0.05.

After FDB fibers had been separated by trituration, mean glycogen content was 48, 52 and 43 μmoles/g dry weight for each of the MEM, MEM +300 μU/mL insulin and 0GLU DMEM conditions, respectively (Figure 1). Glycogen content increased to 86 μmoles/g dry weight for 1 h incubation at 37°C in MEM culture medium supplemented with 10% FBS. It decreased significantly to 47 μmoles/g dry weight during the second hour of incubation. The increase in glycogen content during the first hour of incubation was not affected by the addition 300 μU/mL insulin to the MEM culture medium but the subsequent decrease during the second hour was significantly less in the presence than in the absence of insulin. In glucose‐free DMEM culture medium, glycogen content continued to decrease after trituration reaching 15 μmoles/g dry weight after 1 h and remained low for up to 3 h. So, for the remaining of this study, all [Ca^2+^]_i_ measurements were carried out after single fibers had incubated for at least 1 h and to a maximum of 3 h in MEM medium supplemented with 300 μU/mL insulin or glucose‐free DMEM.

#### 6.1.2 Contraction threshold

**FIGURE 2:**
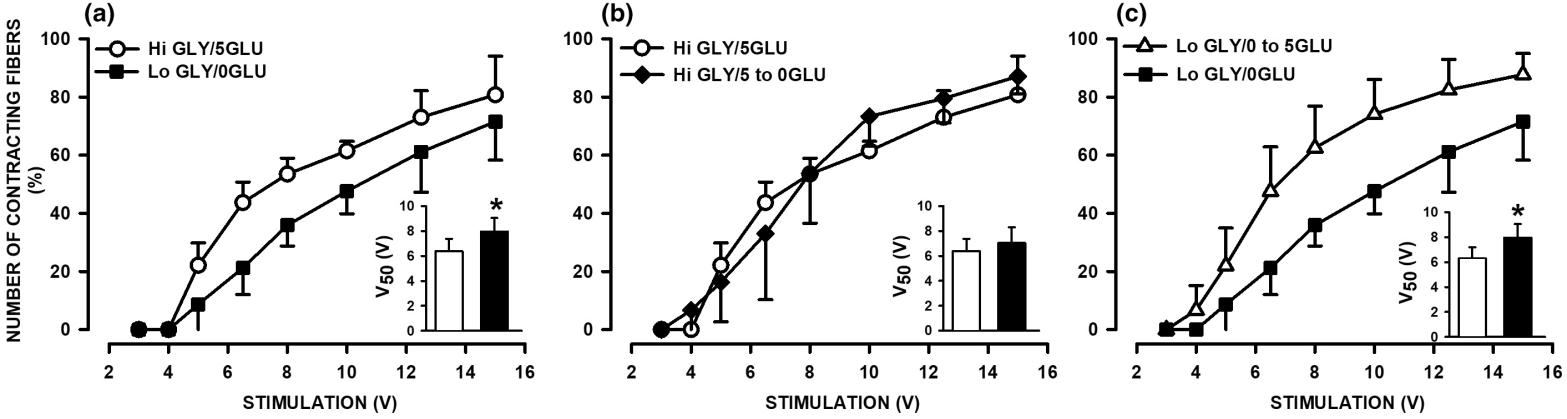
Glycogen depletion significantly shifted the threshold of contracting FDB fibers to higher stimulation strength, an effect reversed by the addition of extracellular glucose. Following fiber dispersion by trituration, fibers were incubated in either glucose containing MEM supplemented with 300 μU/mL insulin to maintain high glycogen content (Hi GLY) or in DMEM lacking glucose also supplemented with 300 μU/mL insulin to deplete glycogen content (Lo GLY) as shown in Figure 1. The number of contracting fibers was measured by counting the number of fibers that shortened and is expressed as a percent of the total number of fibers present in the chamber, which on average was 28 ± 6 fibers (mean ± SD). Measurements were carried out as follows: (a) Hi GLY fibers were tested in 5.5 mM glucose containing physiological solution (Hi GLY/5GLU) while Lo GLY fibers were tested in glucose‐free solution (Lo GLY/0GLU); (b) Hi GLY fibers were tested in glucose‐free physiological solution (Hi GLY/5 to 0GLU) and compared to Hi GLY/5GLU fibers; (c) Lo GLY fibers were tested in 5.5 mM glucose containing physiological solution (Lo GLY/0 to 5GLU) and compared to Lo GLY/0GLU fibers. Vertical bars represent the SD of five mice. Bar graph inserts show mean V_50_ for the different conditions. Correlation coefficients for the V_50_ sigmoidal analyses ranged between 0.963 and 0.998. ANOVA for the stimulation strength versus contraction number relationship: GLY/GLU condition × Stimulation strength interaction *p* = 0.076. *Mean V_50_ (inset) of Lo GLY/0GLU fibers significantly different from that of (a) Hi GLY/5GLU, *t*‐test *p* = 0.035 and (c) Lo GLY/0 to 5GLU, *t*‐test *p* = 0.024.

Next, we determined whether Lo GLY and/or 0GLU affected the contracting threshold. Tetanic contractions were elicited at various stimulus strengths. The number of contracting fibers vs. stimulus strength relationship of Lo GLY‐0GLU fibers was shifted to higher stimulus strength when compared to that of Hi GLY‐5GLU FDB fibers (Figure 2a). At 8 V, 54% of the Hi GLY‐5GLU fibers contracted whereas only 36% of Lo GLY‐0GLU fibers contracted. Mean V_50_, the stimulus strength at which 50% of fibers contracted, was significantly higher for Lo GLY‐0GLU than Hi GLY‐5GLU fibers (Figure 2a, inset).

Removing extracellular glucose from Hi GLY fibers had no significant effect on the number of contracting fibers versus stimulus strength relationship of Hi GLY fibers (Figure 2b and inset). However, exposing Lo GLY fibers to 5GLU 10 min prior to measurements significantly shifted the number of contracting fibers versus stimulus strength relationship to lower stimulus strength (Figure 2c and inset).

#### 6.1.3 Fatigue kinetics of hi GLY‐5GLU FDB fibers

**FIGURE 3:**
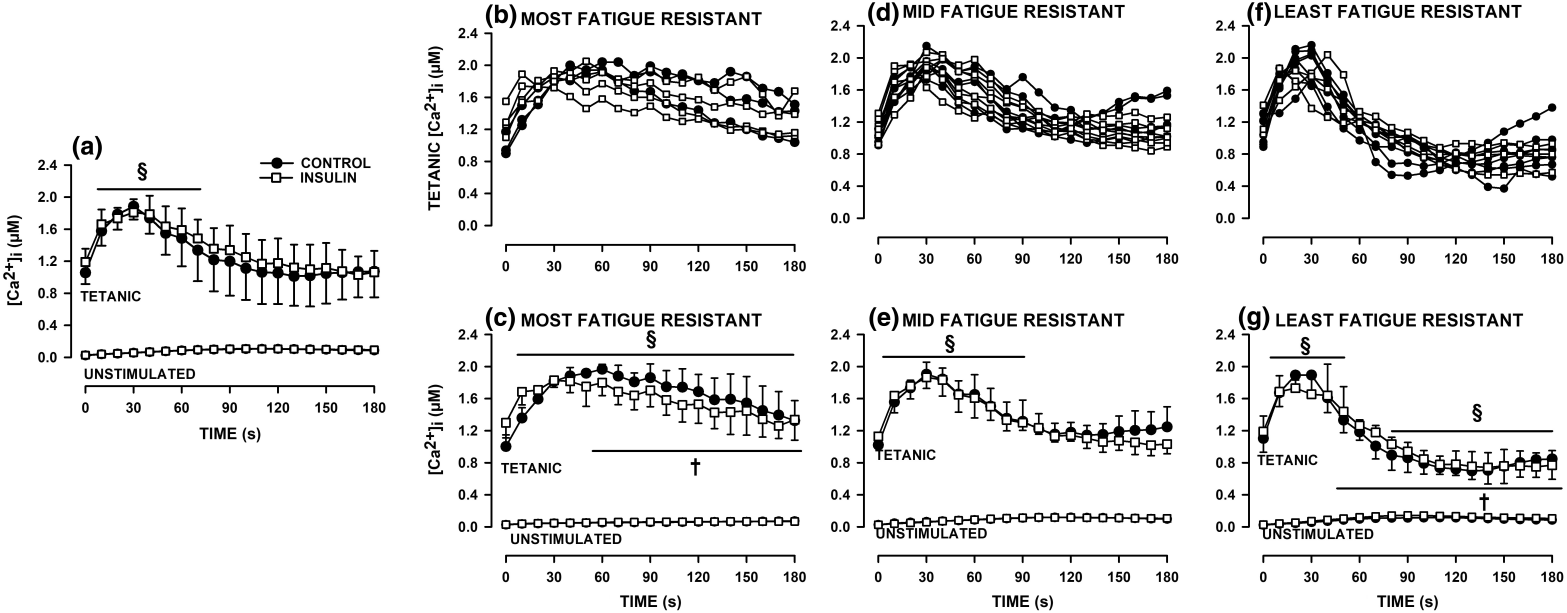
Insulin did not affect how tetanic [Ca^2+^]_i_ changed during fatigue while the decreases in tetanic [Ca^2+^]_i_ were divided into three kinetic groups; that is, the least, mid and most fatigue resistant fibers. Fatigue was elicited with one tetanic contraction every s for 3 min and mean tetanic/unstimulated [Ca^2+^]i are shown every 10 s. (a) Mean tetanic [Ca^2+^]_i_ of all FDB fibers measured under control conditions (i.e., no insulin, *n* = 17 fibers/six mice) and in the presence (*n* = 14/4) of 300 μU/mL insulin. (b) Changes in tetanic [Ca^2+^]_i_ of individual fibers and (c) mean tetanic [Ca^2+^]_i_ for the most fatigue resistant fibers (control *n* = 3 fibers; insulin *n* = 4 fibers). (d) Changes in tetanic [Ca^2+^]_i_ of individual fibers and (e) mean tetanic [Ca^2+^]_i_ for mid fatigue resistant fibers (control *n* = 6 fibers; insulin 7 fibers). (f) Changes in tetanic [Ca^2+^]_i_ of individual fibers and (g) mean tetanic [Ca^2+^]_i_ for the least fatigue resistant fibers (control *n* = 8 fibers; insulin *n* = 3 fibers). Mean unstimulated [Ca^2+^]_i_ are shown in (a, c, e, and f) to demonstrate the extent of the [Ca^2+^]_i_ increases during contractions (see Figure 4 for significant differences in unstimulated [Ca^2+^]_i_). Vertical bars represent the SD. ANOVA: (i) insulin had no significant effect (insulin main effect *p* = 0.372; insulin × time interaction *p* = 0.520; insulin × fatigue resistance interaction *p* = 0.926); (ii) fatigue resistance × time interaction was significant (*p* = 0.0001). ^§^With the horizontal bar shows the time period when mean tetanic [Ca^2+^]_i_ was significantly different from that at 0 s; ^†^With the horizontal bar shows the time period when mean tetanic [Ca^2+^]_i_ was significantly different from that of mid resistant fibers; l.SD, *p* < 0.05.

*Tetanic* [*Ca* ^2+^]_*i*_. Mean tetanic [Ca^2+^]_i_ of all Hi GLY‐5GLU fibers in the absence of insulin significantly increased from 1.06 to 1.90 μM during the first 25 s when fatigue was triggered with one tetanic contraction every s (Figure 3a). Thereafter, tetanic [Ca^2+^]_i_ decreased progressively to 1.11 μM by 105 s, reaching 1.07 μM by the end of the fatigue bout, a value not significantly different from pre‐fatigue value. Insulin, at 300 μU/mL, did not significantly altered the changes in tetanic [Ca^2+^]_i_ during fatigue.

FDB muscles primarily expressed myosin type I, IIA and IIX (Banas et al., 2011). Accordingly, one expects differences in fatigue kinetics between fibers. Indeed, changes in tetanic [Ca^2+^]_i_ could be divided into three groups (Figure 3b,d,f). For one fiber group, referred here as the most fatigue resistant fibers, mean tetanic [Ca^2+^]_i_ significantly increased from 1.00 to 1.97 μM during the first 60 s. Thereafter it slowly decreased reaching 1.33 μM by the end of the fatigue bout; notably, tetanic [Ca^2+^]_i_ remained significantly above the pre‐fatigue tetanic [Ca^2+^]_i_ throughout the entire fatigue bout (Figure 3c). A second fiber group, referred here as mid‐fatigue resistance fibers (Figure 3e), had similar significant initial increases in tetanic [Ca^2+^]_i_ from 1.02 to 1.90 μM but for a much shorter time period, that is 30 s compared to 60 s for the most fatigue resistant fibers. After reaching a peak, decreases in tetanic [Ca^2+^]_i_ for the mid‐resistant fibers was faster compared to the most fatigue resistant fibers, being 0.68 ± 0.06 and 0.31 ± 0.09 μM/min, respectively. The decline in tetanic [Ca^2+^]_i_ for the mid‐fatigue resistant fibers became much slower after 120 s. Mean tetanic [Ca^2+^]_i_ reached 1.25 μM by the end of fatigue, a level not significantly different from that at time 0 s. Mean tetanic [Ca^2+^]_i_ for the third group, referred here as the least fatigue resistant fibers (Figure 3g), reached 1.97 in 25 s. Thereafter, it had the fastest declined rate of 1.34 ± 0.11 μM/min until 120 s. At the end of the fatigue bout, mean tetanic [Ca^2+^]_i_ was 0.85 μM, a value significantly less than at time 0 s.

**FIGURE 4:**
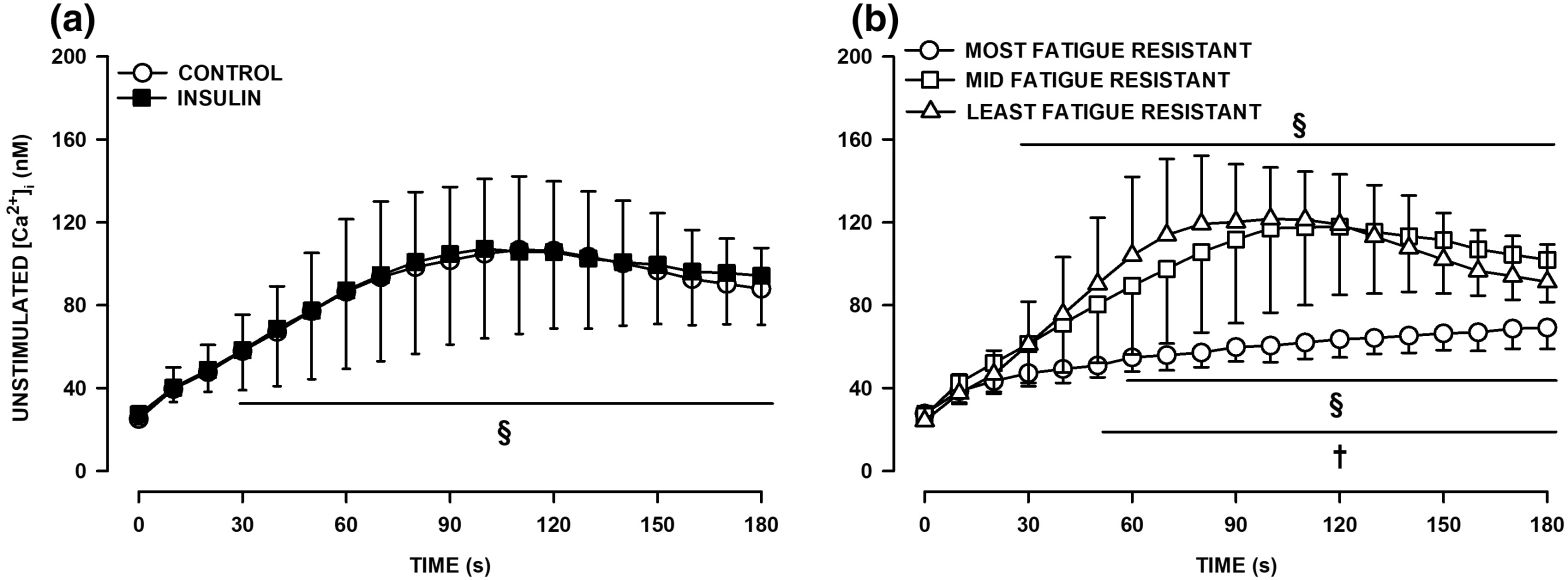
Insulin did not affects how unstimulated [Ca^2+^]_i_ changed during fatigue while the increases in unstimulated [Ca^2+^]_i_ were in the order of most ~ mid > least fatigue resistant fibers. Fatigue was elicited with one tetanic contraction every s for 3 min and mean tetanic/unstimulated [Ca^2+^]i are shown every 10 s. (a) Mean unstimulated [Ca^2+^]_i_ measured under control conditions (i.e., no insulin, *n* = 17 fibers/six mice) and in the presence (*n* = 14/4) of 300 μU/mL insulin. ANOVA: Insulin had no significant effect (main effect *p* = 0.472, interaction with time *p* = 1.000, interaction with fatigue resistance *p* = 0.367). (b) Mean unstimulated [Ca^2+^]_i_ for the most (*n* = 7 fibers/10 mice), mid (*n* = 13 fibers/10 mice) and least (*n* = 11 fibers/10 mice) fatigue resistant FDB fibers (data from control and insulin‐exposed FDB fibers were combined). ANOVA: Fatigue resistance × time interaction was significant (*p* = 0.0001). Vertical bars represent the SD. ^§^With the horizontal bar shows the time when mean unstimulated [Ca^2+^]_i_ was significantly different from that at Time 0 s; ^†^With the horizontal bar shows the time when mean unstimulated [Ca^2+^]_i_ of the most fatigue resistant fibers were significantly different from that of mid fatigue resistant fibers; L.S.D., *p* < 0.05.

Mean unstimulated [Ca^2+^]_i_ of all FDB fibers significantly increased from 25 to 107 nM during the first 115 s before declining to 88 nM by the end of fatigue (Figure 4a). As insulin had no significant effect (Figure 4a), the data from all fibers tested in the absence and presence of insulin were combined to determine differences in unstimulated [Ca^2+^]_i_ between the three fiber groups (Figure 4b). The most fatigue resistant fibers generated the lowest increase in unstimulated [Ca^2+^]_i_ from 28 to 69 nM. The mid‐fatigue resistant and fatigable fibers had similar increases in unstimulated [Ca^2+^]_i_; the values being from 27 nM to 118 nM before it decreased to 102 nM by the end of the fatigue bout for mid‐fatigue resistant fibers, while for the most fatigue resistant fibers the values were 24, 122 and 91 nM. These increases in unstimulated [Ca^2+^]_i_ were significantly greater when compared to those of the most fatigue resistant fibers.

#### 6.1.4 Effects of lo GLY and 0GLU on fatigue kinetics

**FIGURE 5:**
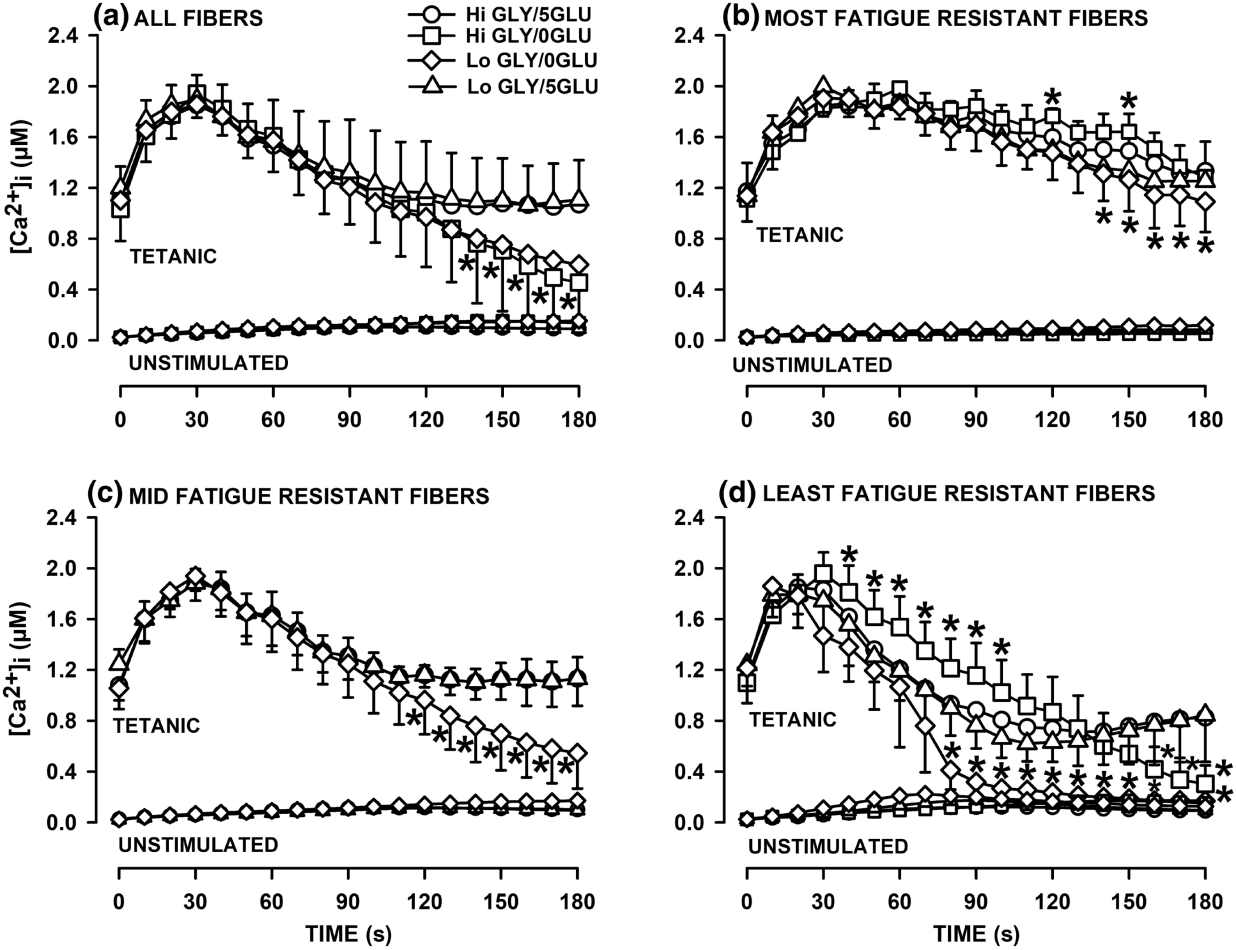
Low glycogen content and the absence of extracellular glucose significantly increased the extent of the tetanic [Ca^2+^]_i_ decrease during the second half of the fatigue period, especially in the most fatigable FDB fibers. Fatigue was elicited with one tetanic contraction every s for 3 min and mean unstimulated [Ca^2+^]i are shown every 10 s. (a) Mean tetanic [Ca^2+^]_i_ of all tested fibers for each of the four experimental conditions. Numbers of fibers/mice were: 31/10, Hi GLY‐5GLU; 13/5, Hi GLY‐0GLU; 23/4, Lo GLY‐5GLU; 25/7, Lo GLY‐0GLU. (b) Mean tetanic [Ca^2+^]_i_ of the most fatigue resistant fibers. Numbers of fibers (number of mice same as in (a): 7, Hi GLY‐5GLU; 2, Hi GLY‐0GLU; 10, Lo GLY‐5GLU; 6, Lo GLY‐0GLU. (c) Mean tetanic [Ca^2+^]_i_ of the mid fatigue resistant fibers. Numbers of fibers: 13, Hi GLY‐5GLU; no fiber found, Hi GLY‐0GLU; 8, Lo GLY‐5GLU; 15, Lo GLY‐0GLU. (d) Mean tetanic [Ca^2+^]_i_ of the most fatigue resistant fibers. Numbers of fibers: 11, Hi GLY‐5GLU; 11, Hi GLY‐0GLU; 5, Lo GLY‐5GLU; 4, Lo GLY‐0GLU. Mean unstimulated [Ca^2+^]_i_ are shown to show the extent of the [Ca^2+^]_i_ increases during contractions (see Figure 6 for significant differences in unstimulated [Ca^2+^]_i_ between experimental conditions). Vertical bars represent the SD. ANOVA: GLY‐GLU condition × Fatigue resistance × Time interaction *p* = 0.001. * Mean tetanic [Ca^2+^]_i_ was significantly different from that at Hi GLY‐5GLU; L.S.D., *p* < 0.05.

Regardless of the GLY‐GLU conditions, insulin had no significant effects on tetanic and unstimulated [Ca^2+^]_i_ (data not shown). So, all measurements in the absence and presence of insulin were combined. When mean tetanic [Ca^2+^]_i_ were calculated from all tested fibers, the changes in tetanic [Ca^2+^]_i_ during fatigue was not affected by a low glycogen content; that is, the fatigue kinetics were the same between Hi GLY‐5GLU and Lo GLY‐5GLU fibers as well as between Hi GLY/0GLU and Lo GLY‐0GLU (Figure 5a). Removing extracellular glucose, on the other hand, significantly altered the fatigue kinetics in the second half of the fatigue bout. That is, 5GLU exposed fibers had a stable tetanic [Ca^2+^]_i_ during the last 60 s whereas tetanic [Ca^2+^]_i_ of 0GLU fibers continued to decrease until the end of the fatigue bout reaching means of 0.46 (Hi GLY/0GLU) and 0.60 μM (Lo GLY/0GLU), which were significantly lower than 1.07 (Hi GLY/5GLU) and 1.11 μM (Lo GLY/5GLU).

As for the Hi GLY‐5GLU fibers, it was also possible to separate the fatigue kinetics into three major groups for the other three experimental conditions. For the most fatigue resistant fibers, there were few significant differences as the changes in tetanic [Ca^2+^]_i_ between the four fiber groups were basically similar for most of the fatigue bout (Figure 5b). The only noticeable differences were mean tetanic [Ca^2+^]_i_ of Lo GLY‐0GLU fibers becoming significantly less than those of Hi GLY‐5GLU fibers during the last 40 s of fatigue. For the mid‐resistant fibers, Lo GLY‐0GLU fibers had the greatest decreases in tetanic [Ca^2+^]_i_ as they became significantly lower than those of Hi GLY‐5GLU for the last 70 s of fatigue (Figure 5c). However, Lo GLY had no effect when extracellular glucose was present; that is, Hi GLY‐5GLU and Lo GLY‐5GLU had similar changes in tetanic [Ca^2+^]_i_ during fatigue.

The greatest effect of Lo GLY and 0GLU was observed with the least fatigue resistant fibers (Figure 5d). First, compared to Hi GLY‐5GLU fibers for which tetanic [Ca^2+^]_i_ reached a steady state after 120 s, the decreases in tetanic [Ca^2+^]_i_ in Hi GLY‐0GLU fibers were continuous until the end of the fatigue bout with almost no increase in tetanic [Ca^2+^]_i_ upon contraction at the very end of the fatigue. Second, compared to Hi GLY‐0GLU fibers, the decrease in tetanic [Ca^2+^]_i_ was the fastest and greatest in Lo GLY‐0GLU fibers with almost no more increase in tetanic [Ca^2+^]_i_ upon contractions after 90 s. One interesting observation was the lack of any effects of Lo GLY when extracellular glucose was present; that is, for all three fiber groups, the changes in tetanic [Ca^2+^]_i_ during fatigue was not different between Hi GLY‐5GLU and Lo GLY‐5GLU.

**FIGURE 6:**
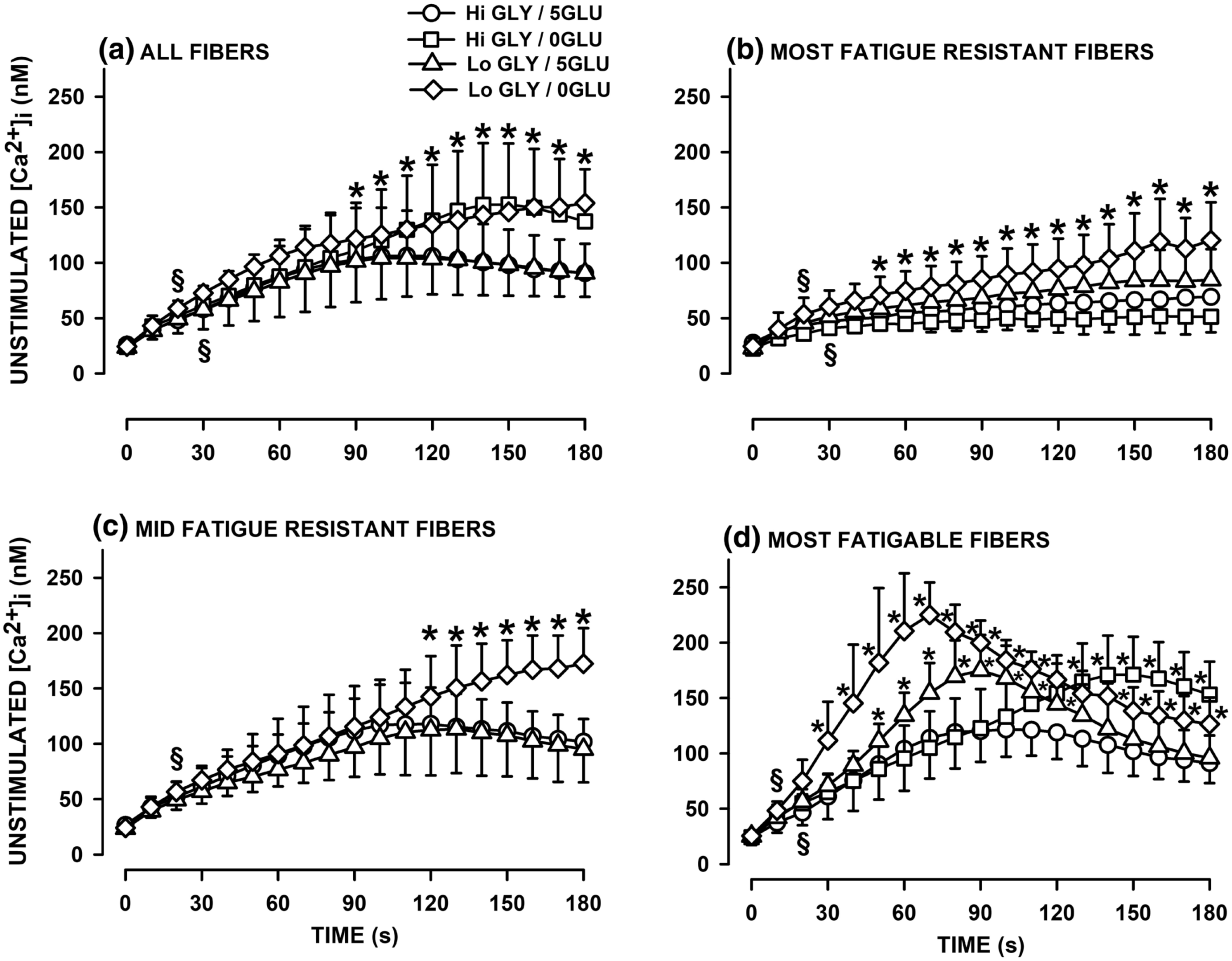
Low glycogen content and the absence of extracellular glucose significantly resulted in greater increases in unstimulated [Ca^2+^]_i_ during fatigue period, especially in the most fatigable FDB fibers. Fatigue was elicited with one tetanic contraction every s for 3 min and mean unstimulated [Ca^2+^]i are shown every 10 s. (a) Mean unstimulated [Ca^2+^]_i_ of all tested fibers for each of the four experimental conditions. (b) Mean unstimulated [Ca^2+^]_i_ of the most fatigue resistant fibers. (c) Mean unstimulated [Ca^2+^]_i_ of the mid fatigue resistant fibers. (d) Mean unstimulated [Ca^2+^]_i_ of the most fatigue resistant fibers. Vertical bars represent the SD. Numbers of fibers/mice are as indicated in Figure 5. ANOVA: GLY/GLU condition × Fatigue resistance × Time interaction *p* = 0.0001. ^§^Indicate the time when mean unstimulated [Ca^2+^]_i_ became significantly different from that at Time 0 s; *Mean unstimulated [Ca^2+^]_i_ was significantly different from that at 5GLU/HI GLY; L.S.D., *p* < 0.05.

*Unstimulated* [*Ca* ^2+^]_*i*_. In the presence of extracellular glucose and regardless of the glycogen content, mean unstimulated [Ca^2+^]_i_ from all tested fibers significantly increased from 25 nM to 105 during the first 100 s decreasing slightly to 91 nM by the end of the fatigue bout (Figure 6a). In the absence of extracellular glucose and regardless of the glycogen content, unstimulated [Ca^2+^]_i_ continuously and significantly increased throughout the fatigue period from 24 nM to 137 for Hi GLY‐0GLU fibers and from 25 to 154 nM for Lo GLY‐0GLU fibers.

The most fatigue resistant fibers had the lowest increases in unstimulated [Ca^2+^]_i_ (Figure 6b). For these fibers, the increases in unstimulated [Ca^2+^]_i_ was basically the same for Hi GLY‐5GLU and Hi GLY‐0GLU from 28 to 69 nM for the former and from 23 to 51 nM for the latter. Lo GLY fibers had greater increases in unstimulated [Ca^2+^]_i_, especially in the absence of extracellular glucose; being from 23 to 25 nM to 85 nM for Lo GLY‐5GLU fibers and 120 nM for Lo GLY/0GLU fibers. For the mid‐resistant fatigue fibers, unstimulated [Ca^2+^]_i_ increased steadily for 120 s from 24 to 27 nM to 113–118 nM when extracellular glucose was present with no difference in regard to glycogen content (Figure 6c). Subsequently, unstimulated [Ca^2+^]_i_ decreased reaching 95–102 nM by the end of fatigue. The increase in unstimulated [Ca^2+^]_i_ was significantly greater for Lo GLY‐0GLU reaching 172 nM by the end of fatigue. The most fatigable fibers had the greatest increases in unstimulated [Ca^2+^]_i_ (Figure 6d). For Hi GLY fibers, unstimulated [Ca^2+^]_i_ increased similarly for 90 s in the absence and presence of extracellular glucose. Thereafter, unstimulated [Ca^2+^]_i_ decreased and reached 91 nM for Hi GLY‐5GLU fibers but continued to increase for Hi GLY‐0GLU reaching a maximum of 172 nM at 155 s. The initial increases in unstimulated [Ca^2+^]_i_ was significantly greater in Lo GLY fibers reaching a peak of 175 nM at 90 s in the presence of extracellular glucose and 225 nM at 70 s in the absence of extracellular glucose. Thereafter unstimulated [Ca^2+^]_i_ largely decreased at a time when tetanic [Ca^2+^]_i_ became very small (Figure 5d).

### 6.2 FDB bundles

**FIGURE 7:**
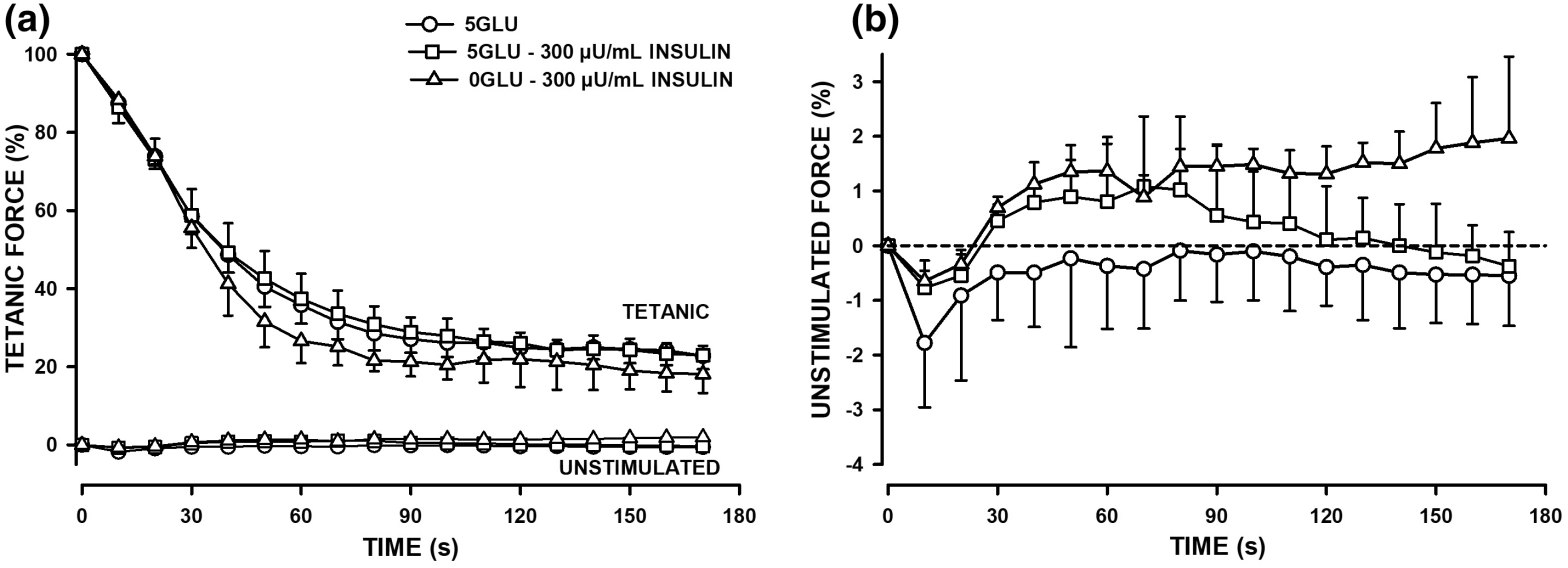
Removing extracellular glucose caused a slight but insignificant greater decrease in tetanic force decrease while insulin had no effect in FDB bundles. (a) Mean tetanic and (b) unstimulated force. All FDB bundles with normal glycogen content (see Figure 8) were exposed to solutions containing 5.5 mM glucose in the absence or in the presence of 300 μU/mL insulin or in glucose‐free solution containing 300 μU/mL insulin 60 min prior to fatigue. Fatigue was elicited with one 200 ms contraction every s for 3 min (data are shown at every 10 s). Tetanic and unstimulated forces are expressed as a percent of the tetanic force at time 0 s. Vertical bars represent the SD. of 3–5 FDB bundles. ANOVA: GLU conditions × Time interaction for tetanic force and unstimulated force *p* values were 0.89 and 0.99, respectively; *p* values for GLU treatment main effect were 0.76 and 0.29, respectively.

During fatigue, tetanic force of FDB bundles exposed to 5.5 mM glucose decreased by 67% in the first 30 s, being just 22% of pre‐fatigue mean force by 180 s (Figure 7a). The addition of 300 μU/mL insulin had no significant effect on the decrease in tetanic force. Cairns and Renaud (2023) recently reported no effect of Lo GLY on the fatigue kinetics in mouse soleus, so this condition was not done here. The removal of extracellular glucose resulted in slightly but insignificant greater decreases in tetanic force between 50 and 100 s. Unstimulated force did not change significantly during fatigue and neither insulin nor the lack of glucose had a significant effect (Figure 7b).

**FIGURE 8:**
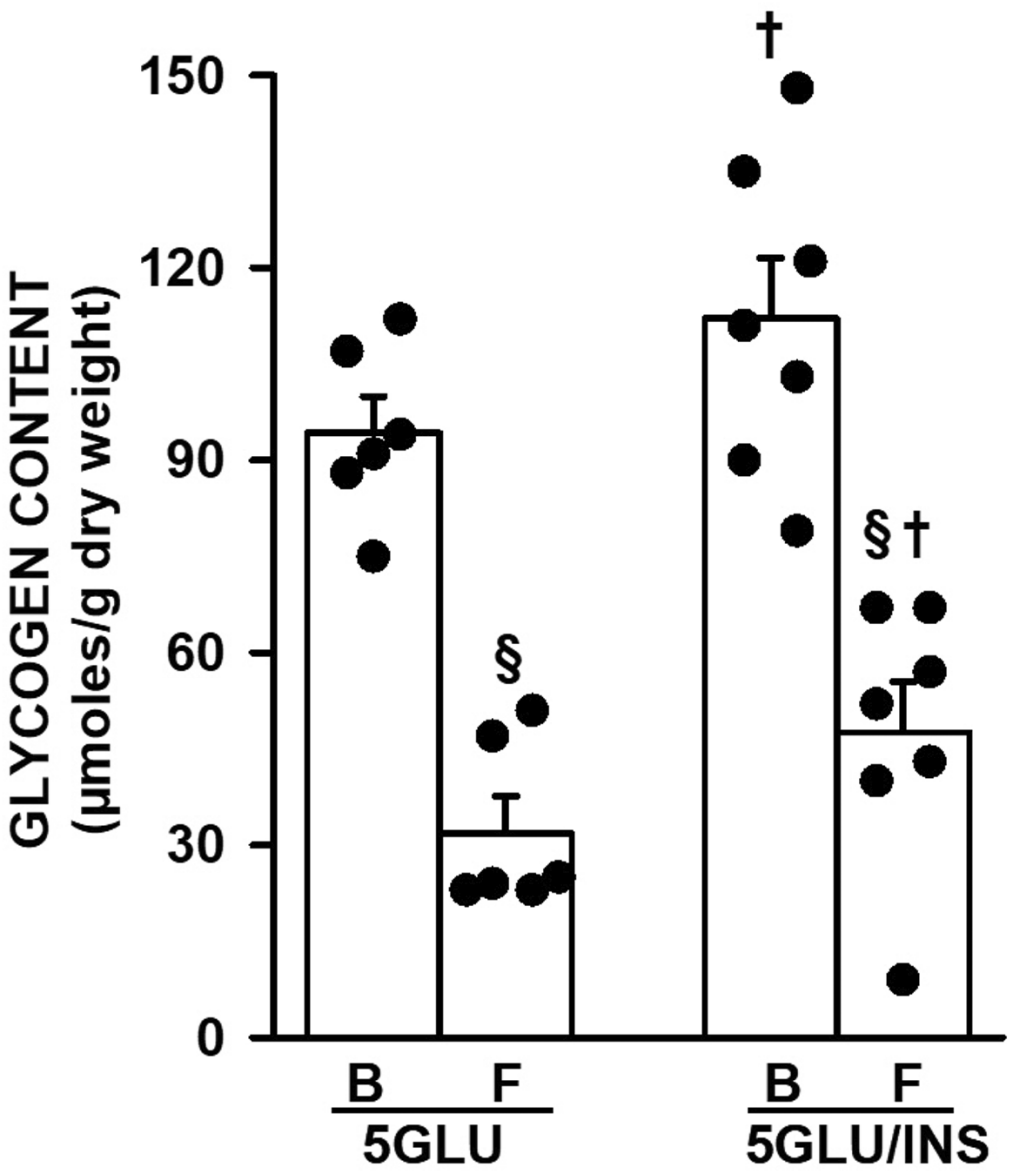
Insulin‐exposed FDB bundles had significantly greater glycogen content but did not significantly affect the extent of glycogen utilization during fatigue. Fatigue was elicited with one tetanic contraction every s for 3 min. Paired FDB bundles were used; one bundle was frozen before fatigue and the other immediately after fatigue. B, before fatigue; F, after fatigue; 5GLU, 5,5 mM glucose; INS, 300 mU/mL insulin. Vertical bars represent the SD. of 5 FDB bundles. ANOVA: Insulin condition × Fatigue time interaction *p* = 0.885, main effect *p* values for insulin condition and time were 0.033 and 0.0001, respectively. ^§^Mean glycogen content at the end of fatigue significantly different from that before fatigue; ^†^Mean glycogen content of insulin‐exposed FDB was significantly different from that of control; L.S.D., *p* < 0.05.

Insulin‐exposed FDB muscles had a significantly greater glycogen content than control FDB prior to and at the end of fatigue. However, the amount of glycogen used during fatigue was not different between control and insulin‐exposed FDB bundles (Figure 8).

## 7 DISCUSSION

The objective of this study was to determine the importance of glucose and glycogen with an approach to deplete glycogen store by exposing FDB fibers to glucose‐free solutions and not following fasting or fatigue/recovery processes. The major findings of this study were as follows. (1) Glycogen levels in FDB single fibers exposed one h in glucose free DMEM had only 19% of the glycogen content of fibers exposed to glucose‐containing MEM medium supplemented with 300 μU/mL insulin. (2) The number of contracting fibers versus stimulation strength relationship of Lo GLY‐0GLU single FDB fibers was significantly shifted to higher stimulus strength compared to Hi GLY‐5GLU fibers. (3) The shift was reversed when glucose was added to Lo GLY‐0GLU fibers whereas the removal of glucose for Hi GLY‐5GLU fibers had no effect. (4) When tested at 37°C and based on the changes in tetanic [Ca^2+^]_i_, the fatigue kinetics of single FDB fibers could be divided into three major groups: the most, mid and the least fatigue resistant fibers. (5) Low glycogen content and removal of extracellular glucose augmented the rate at which tetanic [Ca^2+^]_i_ decreased during fatigue in the order of the least > mid > most fatigue resistant fibers. (6) Unstimulated [Ca^2+^]_i_ increased during fatigue and the extent of the increase was in the order of the least > mid > most fatigue resistant fibers. (7) Lo GLY and 0GLU caused greater increases in unstimulated [Ca^2+^]_i_ in the order of the least > mid > most fatigue resistant fibers.

## 8 CARBOHYDRATE EFFECTS: FDB BUNDLES VERSUS SINGLE FIBERS

For mouse FDB bundles, 0GLU caused slightly greater but insignificant decreases in tetanic force whereas in single fibers it significantly and largely increased the extent of the tetanic [Ca^2+^]_i_ decreases; that is, FDB single fibers were more sensitive to extracellular glucose than FDB bundles. O_2_ and glucose diffusion limitation is most likely responsible for the difference between FDB bundles and single fibers. Based on an O_2_ diffusion model at 37°C (Barclay, 2005)‚ no hypoxic condition occurs in 5–6 mg mouse soleus muscles so that muscles maintain tetanic force over period as long as 80 min (Lucas et al., 2014; Uwera et al., 2020). However, increasing contraction frequency to once every s leads to anoxic/hypoxic core as O_2_ diffusion from the surface to the muscle middle core is too slow even with 1–2 mg FDB bundles as used in this study. In fact, mouse soleus muscles fatigued faster than soleus single muscle fibers because of a lack of O_2_ availability throughout the soleus (Zhang et al., 2006), and the same is true for FDB bundles and single fibers. Like for the limited O_2_ diffusion, the same is expected for glucose diffusion. In other words, the glucose availability from the interstitial space for FDB bundles may not be very different during fatigue whether or not extracellular glucose is present in the physiological solution resulting in an apparent lack of a glucose effect with FDB bundles.

This O_2_ and glucose diffusion limitation does not occur with single fibers and thus single muscle fibers are a better preparation to study the effect of extracellular glucose. Furthermore, the greater extent of fatigue in the absence of extracellular glucose in FDB is in agreement with studies in which rat plantaris and soleus muscles were used in situ (Karelis et al., 2002, 2003; Marcil et al., 2005). Together these two studies and this one support the previous suggestion that studying fatigue using single fibers better reflects what is happening with skeletal muscles in situ (Zhang et al., 2006).

Finally and notably, the method used to calculate changes in tetanic [Ca^2+^]_i_ during fatigue had an impact on how Lo GLY and 0GLU effects are observed. A glucose effect was clearly observed whether tetanic [Ca^2+^]_i_ values were averaged for all fibers tested (Figure 5a) or separated into three groups according to the fatigue kinetics (Figure 5b–d). The same did not apply for Lo GLY. That is, there was no evidence for a Lo GLY effect when tetanic [Ca^2+^]_i_ were average from all tested fibers (Figure 5a), but was observed when fatigue kinetics were separated into three groups (Figure 5c,d). Furthermore, the extent of the Lo GLY and 0GLU effects on both tetanic and unstimulated [Ca^2+^]_i_ were different between fibers with different fatigue resistance. So, the remaining discussion will be based on the results obtained from the three major fatigue kinetic groups.

## 9 CARBOHYDRATE AVAILABILITY BUT NOT INSULIN AFFECTS FATIGUE KINETICS

Insulin did not alter fatigue kinetics in single fibers. This is in agreement with a study in which a hyperinsulinemic‐euglycemic condition did not affect the change in tetanic force of rat plantaris muscle indirectly stimulated when compared to control (Karelis et al., 2003). Also, insulin did not affect the extent of the glycogen depletion, which is in agreement with another study involving rat soleus (Marcil et al., 2005).

In this study, a Lo GLY model was generated in collagenase‐dispersed single muscle fibers without a previous fatigue bout and recovery in 0GLU, for which fibers did not fully recover prior to the second fatigue bout, or overnight fasting, for which hormonal changes may affect muscle responses to fatigue. Although the glycogen content was low following the trituration of FDB muscle fibers, 1 h incubation in glucose‐containing MEM culture medium supplemented with 300 μU/mL insulin was sufficient for the glycogen content of FDB single fibers to increase toward a content similar to that observed in isolated mouse muscles (Barnes et al., 2001; Helander et al., 2002; Okamoto et al., 2001) as well as in situ mouse skeletal muscles (Manchester et al., 1996). Furthermore, glycogen content remained elevated for at least another 2 h. One h incubation in 0GLU DMEM culture medium depleted glycogen reserve to 19% of the Hi GLY content, which approximated values reported at exhaustion (i.e., when the workload could no longer be maintained) during exercise in human, that is, 5%–10% (Bergström et al., 1967; Hermansen et al., 1967). Thus, the extent of the glycogen depletion in FDB single fibers is representative of the glycogen depletion reported in human at exhaustion.

Cairns and Renaud (2023) reported large activation of AMPK when the glycogen content of mouse soleus muscle had been depleted by 70% following incubation in 0GLU. As our Lo GLY fibers had 81% less glycogen than in situ mouse muscles, it can be expected that Lo GLY fibers also had large increase in AMPK activity, suggesting some sort of metabolic stress. However, neither Lo GLY nor 0GLU affected pre‐fatigue tetanic [Ca^2+^]_i_, which is in agreement with the lack of effect of Lo GLY or 0GLU on tetanic force, resting E_M_ and action potential in soleus muscles when [K^+^]_e_ is 4 mM (Cairns & Renaud, 2023). Thus, regardless of the nature of the metabolic stress, Lo GLY‐0GLU FDB fibers were capable of fully contracting prior to fatigue. This is different from studies in which glycogen was depleted with one fatigue bout and recovery in 0GLU, a condition for which force and Ca^2+^ transient had not fully recovered prior to the second fatigue bout; that is, fibers were still in a fatigue mode prior to the second fatigue bout (Chin & Allen, 1997). Lo GLY/0GLU, however, caused a shift toward greater stimulation strength to trigger contractions in unfatigued FDB fibers when compared to Hi GLY/5GLU. A similar shift was not observed with Hi GLY fibers following the removal of glucose whereas a reverse shift was observed when glucose was re‐introduced to Lo GLY fibers. We therefore suggest that both glycogen and glucose availability alters contraction threshold and that the Lo GLY effect can be compensated when glucose transport is high (see section entitled “The lack of glycogen effect when extracellular glucose is present” for more details about the glucose compensation).

### 9.1 Glycogen and glucose availability affects fatigue kinetics to various degrees among FDB fibers

Decreases in glycogen and glucose availability significantly increases the rate of fatigue measured from the decrease in tetanic [Ca^2+^]_i_ while it caused greater increases in unstimulated [Ca^2+^]_i_. This is in agreement with studies demonstrating that glucose supplementation has the reverse effect; that is, it reduces the extent of the force decrease during fatigue (Green et al., 2007; Karelis et al., 2002, 2003; Marcil et al., 2005; Stewart et al., 2007). More importantly, this study demonstrates distinct differences between fibers. For the most fatigue resistant fibers, Lo GLY and/or 0GLU barely affected changes in tetanic [Ca^2+^]_i_, while causing greater unstimulated [Ca^2+^]_i_ during the last 30 s of the fatigue bout. Mid fatigue resistant fibers and the least fatigue resistant fibers were affected by Lo GLY and/or 0GLU, being the greatest for the least fatigue resistant group. For both groups, Lo GLY did not affect how tetanic [Ca^2+^]_i_ changed during fatigue when extracellular glucose was present. In the absence of extracellular glucose, on the other hand, tetanic [Ca^2+^]_i_ never reached a steady state. For Hi GLY/0GLU fibers, the decrease in tetanic [Ca^2+^]_i_ of the least fatigue resistant fibers continued until the end of the fatigue bout with an almost complete cessation of contraction. For Lo GLY/0GLU fibers, the decrease in tetanic [Ca^2+^]_i_ of mid‐fatigue resistance fibers was continuous until the end of the fatigue bout while for the least fatigue resistant fibers the decrease in tetanic [Ca^2+^]_i_ was continuous until contraction completely stopped by 90 s. Thus the effect of Lo GLY and 0GLU on the extent of fatigue is in the order of the least > mid > most fatigue resistant fibers. Glycogen and glucose availability also affected unstimulated [Ca^2+^]_i_. Similar to the tetanic [Ca^2+^]_i_, the effects on unstimulated [Ca^2+^]_i_ were observed in all fatigue resistant groups in the order of the least > mid > most fatigue resistant fibers.

### 9.2 Carbohydrate availability affects fatigue kinetics in the order of type IIX>IIA>I

In FDB muscle, type I, I‐IIA, and I‐IIX fibers constitute 16% of all fibers while type IIA, IIA‐IIX and IIX fibers respectively constitute 19%, 32% and 21% (the remaining 12% being a complex combination of myosin isoforms, including I‐IIA‐IIX) (Banas et al., 2011). Despite this complex fiber type distribution, only three distinct fatigue kinetics were discerned based on how both tetanic and unstimulated [Ca^2+^]_i_ changed during fatigue. It is well established that fatigue resistance is in the order of type I > IIA > IIX. We therefore suggest that the most fatigue resistant fibers observed in this study were fibers primarily expressing type I (alone or in combination with IIA and IIX), the least fatigue resistant being fibers primarily expressing type IIX myosin and the mid‐resistant fibers type IIA fibers. This then implies that type I fibers are the least affected by Lo GLY and/or 0GLU while type IIX fibers are the most affected. A greater glucose effect on fatigue resistance in type II than in I fibers has also been reported in rat muscles that were indirectly stimulated for 60 min to trigger fatigue; that is, glucose infusion reduced the extent of fatigue by 25% in rat soleus (95% type I fibers), and only by 15% in plantaris muscles (94% type II fibers) (Karelis et al., 2002; Marcil et al., 2005). We therefore suggest that the dependence of fibers on glycogen and glucose availability to sustain muscle activity is in the order of type IIX > IIA > I fibers.

One reason for the difference in the glycogen and glucose effects between fiber types is the ATP demand being in the order of type I < IIA < IIX fiber. This is because compared to type II fibers, type I fibers have (i) lower NKA activity as Na^+^ influx and K^+^ efflux during action potentials is 6‐times smaller (Clausen et al., 2004; Delp & Duan, 1996); (ii) 3‐times less Ca^2+^ ATPase pump activity (Dufresne et al., 2016); (iii) 2.0–2.5 times less myosin ATPase activity (Essen et al., 1975). A second reason for the differences in glycogen and glucose effects is the greater oxidative capacity and greater triglyceride storage in type I than II fibers (Baldwin et al., 1973; Essen et al., 1975; Reitman et al., 1973). This difference is possibly amplified by the increase in AMPK activity in Lo GLY fibers, which then increases glucose uptake and fatty acid oxidation (Fujii et al., 2006; Lee et al., 2006; Merrill et al., 1997).

### 9.3 The lack of glycogen content effect when extracellular glucose is present

The lack of a glycogen effect in the presence of extracellular glucose on contraction threshold in unfatigued fibers and decrease in tetanic [Ca^2+^]_i_ of mid‐resistant and least fatigue resistant fibers during fatigue was initially surprising. A first explanation would be that glycogen availability is not important for short highly intense fatigue bout like the one used in this study. However, this is unlikely for two reasons. First, for the least fatigue resistant fibers, the decreases in tetanic [Ca^2+^]_i_ and increases in unstimulated [Ca^2+^]_i_ were greater for Lo GLY/0GLU fibers than for Hi GLY/0GLU fibers. If glycogen content had no effect, then there should have been no differences between the two experimental conditions as observed when all data were averaged as in Figure 5a.

Second, as mentioned above a large AMPK activation when glycogen stores are depleted is expected to increase glucose transport. Perhaps, considering the large glucose effect on contraction threshold and tetanic [Ca^2+^]_i_. re‐introduction of glucose to Lo GLY fibers 10 min prior to fatigue may have been sufficient to provide sufficient glucose for ATP generation, compensating for the lack of glycogen. We therefore suggest that during a 3 min fatigue bout elicited with one tetanic contraction every s the decrease in tetanic [Ca^2+^]_i_ and thus tetanic force become faster and greater when either/both glycogen and glucose availability is low and that extracellular glucose can compensate for Lo GLY.

## 10 MECHANISMS FOR THE CARBOHYDRATE AVAILABILITY EFFECTS

Glucose supplement has been shown to protect membrane excitability during fatigue bout (Karelis et al., 2002; Stewart et al., 2007). The activity of at least three cell membrane components are ATP dependent: NKA, K_ATP_ and ClC‐1 Cl^−^ channels (Noma, 1983; Tseng et al., 2007; Zhang et al., 2008). Moreover, the activity of the first two components is dependent on glycolytic ATP generation (Dhar‐Chowdhury et al., 2007; James et al., 1996; Okamoto et al., 2001; Weiss & Lamp, 1989). Finally, glucose supplementation during fatigue increases in maximum NKA activity (Green et al., 2007), while the lack of glucose causes sooner and greater activation of K_ATP_ and ClC‐1 channels (Pedersen, de Paoli, et al., 2009; Pedersen, Macdonald, et al., 2009). So, lower glycogen and/or glucose availability is expected to reduce NKA activity, allowing greater increases in [K^+^]_e_ and [Na^+^]_i_. which then reduces membrane excitability (Cairns et al., 2003, 2022; Cairns & Renaud, 2023), while greater activation of K_ATP_ and ClC‐1 channels reduces action potential amplitude (Gong et al., 2003; Pedersen, de Paoli, et al., 2009; Pedersen, Macdonald, et al., 2009).

Glycogen and glucose availability also affects Ca^2+^ release and uptake. Glycogen particles and glycolytic enzymes are associated with sarcoplasmic reticulum (Nielsen et al., 2009; Xu & Becker, 1998). There is evidence for lower Ca^2+^ release and uptake rates by sarcoplasmic reticulum during fatigue and to a greater extent when glycogen content is low (Duhamel et al., 2006a, 2006b; Gejl et al., 2014; Krustrup et al., 2011; Ørtenblad et al., 2011). Finally, slower Ca^2+^ reuptake at Lo GLY and/or 0GLU is probably the major reason for the greater increase in unstimulated [Ca^2+^]_i_ during fatigue when compared to Hi GLY‐5GLU condition.

In conclusion, in this study (i) a new approach was used to study the effect of glycogen depletion during contractile activity leading to fatigue and (ii) the interactive effects of low glycogen and/or glucose availability was studied. Testing single FDB fibers at 37°C, we showed that low glycogen and glucose separately and together increases contraction threshold in unfatigued fibers, accelerates the decrease in tetanic [Ca^2+^]_i_ and increases in unstimulated [Ca^2+^]_i_ during a fatigue bout triggered by one contraction every s for 3 min. The glycogen and glucose dependency during fatigue was in the order of the least (type IIX fibers) > mid (type IIA fibers) > the most (type I fibers) fatigue resistant fibers.

## CONFLICT OF INTEREST STATEMENT

The authors have no conflict of interest to declare.

## ETHICS STATEMENT

Care for mice were according to the guidelines of the Canadian Council for Animal Care (CCAC); and, with the approval of The Animal Care Committee of the University of Ottawa. None of the data in this paper has been published elsewhere.
